# PMAVP: A Mamba-Inspired Deep Learning Framework for Antiviral Peptide Identification and Functional Activity Prediction

**DOI:** 10.3390/ijms27177764

**Published:** 2026-08-30

**Authors:** Peiwei Wei, Weihao Su, Qingsong Qin, Chuliang Wei, Yi Shi, Guishan Zhang

**Affiliations:** 1 Laboratory of Human Virology and Oncology, Shantou University Medical College, Shantou 515041, China; peiwei_wei@163.com (P.W.); qsqin@stu.edu.cn (Q.Q.); 2 Joint Shantou International Eye Center of Shantou University and the Chinese University of Hong Kong, Shantou 515041, China; 3 Department of Electronic Engineering, College of Engineering, Shantou University, Shantou 515063, China; 23whsu@stu.edu.cn (W.S.); clwei@stu.edu.cn (C.W.); shiyi@stu.edu.cn (Y.S.)

**Keywords:** antiviral peptide, Mamba, multi-task learning, DeepSHAP

## Abstract

Accurate computational prediction of antiviral peptides (AVPs) can accelerate peptide screening and reduce experimental costs. However, existing deep learning-based methods still suffer from severe class imbalance, over-reliance on handcrafted features and limited interpretability. Here, we propose PMAVP, a multi-task learning framework that integrates the ProtT5 pre-trained protein language model with a Mamba-inspired module for AVP identification and functional activity prediction. We use ProtT5 to extract deep semantic representations from peptide sequences and a Mamba module to capture long-range dependencies at a lower computational complexity. We introduce Focal Loss to mitigate class imbalance and leverage transfer learning to enhance performance on functional activity prediction. Experimental results demonstrate that our model achieves superior performance in terms of prediction accuracy, stability, and computational efficiency. Furthermore, DeepSHAP-based interpretability analysis reveals that the first 40 amino acid residues contribute substantially to AVP prediction.

## 1. Introduction

Viral infections remain a serious threat to global public health, human life and social stability [1]. The continuous emergence and mutation of pathogenic viruses have caused substantial medical and economic burdens worldwide [2,3]. Though antiviral drugs have been used in clinical treatment, their effectiveness is limited by viral resistance and potential toxicity [4,5]. Thus, the discovery of novel antiviral agents with high specificity and favorable biological properties has become an important research direction [6]. Antiviral peptides (AVPs) have attracted increasing attention as promising candidates for antiviral drug development [7]. AVPs can inhibit viral infection through multiple mechanisms, such as by blocking viral attachment, preventing membrane fusion, interfering with viral replication, and regulating host immune responses [8]. Compared with traditional small-molecule drugs, AVPs exhibit high sequence diversity, strong target specificity, and relatively low risk of drug resistance. However, experimental identification of AVPs is usually time-consuming, labor-intensive, and expensive. Moreover, predicting the functional activities of AVPs against specific viral families or species is also essential for understanding their antiviral spectrum and supporting downstream therapeutic development [9].

Recently, machine learning and deep learning approaches have been applied to AVP prediction. For instance, Pang et al. [10] proposed AVPIden, which uses feature descriptors and a balanced random forest classifier to identify antiviral peptides. However, machine learning-based methods mainly rely on manually designed sequence descriptors and traditional classifiers. Li et al. [11] proposed DeepAVP, which combines one-hot encoding with convolutional neural networks (CNNs) [12] and bidirectional long short-term memory (BiLSTM) networks [13], enabling the model to automatically capture local sequence motifs and contextual dependencies from peptide sequences. Lin et al. [14] developed AI4AVP by incorporating physicochemical properties into a CNN framework, indicating that biochemical information can provide useful complementary features for AVP identification. Cao et al. [15] proposed FFMAVP, which integrates feature descriptors and sequence encoding with a CNN-BiLSTM architecture to improve peptide representation learning. Guan et al. [16] proposed AVP-IFT, which combines CNN, BiLSTM, and Transformer [17] to learn local patterns and global contextual information.

Although these studies have promoted the development of AVP prediction, several limitations remain. First, data imbalance is a common issue, with non-AVPs greatly outnumbering AVPs in AVP datasets. This issue may bias the model toward the majority class and reduce its ability to identify minority AVP samples. Second, many existing methods rely heavily on manually extracted features, which require additional preprocessing and domain knowledge. Third, although CNN-, BiLSTM-, and Transformer-based models have shown promising performance, their computational efficiency remains a concern. CNN is effective in extracting local motifs but limited in modeling long-range dependencies. BiLSTM can capture sequential information but encounters difficulty in parallelizing efficiently. Transformers improve global dependency modeling through self-attention, but their computational complexity increases substantially with sequence length. Fourth, the interpretability of existing models is still insufficient. Finally, most existing algorithms mainly focus on classification between AVPs and non-AVPs, while the prediction of functional activities, such as antiviral activity against specific viral families or viral species, remains underexplored.

To address these challenges, we propose PMAVP, a multi-task learning framework that integrates the ProtT5 protein pretrained language model [18] with Mamba [19] for AVP identification and functional activity prediction. Specifically, PMAVP can distinguish AVPs from non-AVPs and predict their functional activities against eight viral species—FIV, HIV, HCV, HPIV3, HSV1, INFVA, RSV, and SARS-CoV—and six viral families—Coronaviridae, Retroviridae, Herpesviridae, Paramyxoviridae, Orthomyxoviridae, and Flaviviridae. ProtT5 is used to extract deep semantic representations from peptide sequences, enabling the model to learn informative sequence features from large-scale protein data without relying heavily on handcrafted descriptors. Mamba is introduced to efficiently model long-range dependencies in peptide sequences while maintaining lower computational complexity. Our model uses Focal Loss [20] to increase the contribution of hard-to-classify samples during training to alleviate the data-imbalance issue in AVP datasets, thereby improving the robustness of the model. Furthermore, we apply a transfer learning strategy for functional activity prediction. Specifically, we first pretrain our model for the binary AVP identification task to learn general and discriminative peptide representations. Subsequently, the pretrained parameters are transferred for the functional activity classification task and further fine-tuned using functional activity datasets, allowing the model to adapt to more complex multi-class distributions. Ablation analysis results demonstrate that the Mamba module and global max pooling are key components contributing to the performance improvement of PMAVP. Our model shows satisfactory performance in terms of prediction accuracy, stability and computational efficiency. In addition, DeepSHAP-based interpretability analysis [21] results show the first 40 amino acids of peptide sequences play an important role in AVP prediction, suggesting that this region may contain critical functional information associated with antiviral activity.

## 2. Results

### 2.1. Ablation Study Shows the Importance of Mamba Block

To further investigate the contributions of the core components in PMAVP and validate the rationality of the proposed architecture, a series of ablation experiments were conducted on Dataset 2. By removing or replacing specific functional modules from the original model, five model variants were constructed (Table 1) to quantitatively evaluate the effects of the Mamba module, the dual-pooling strategy, and the Focal Loss function on feature extraction and classification performance.

**Figure 1:**
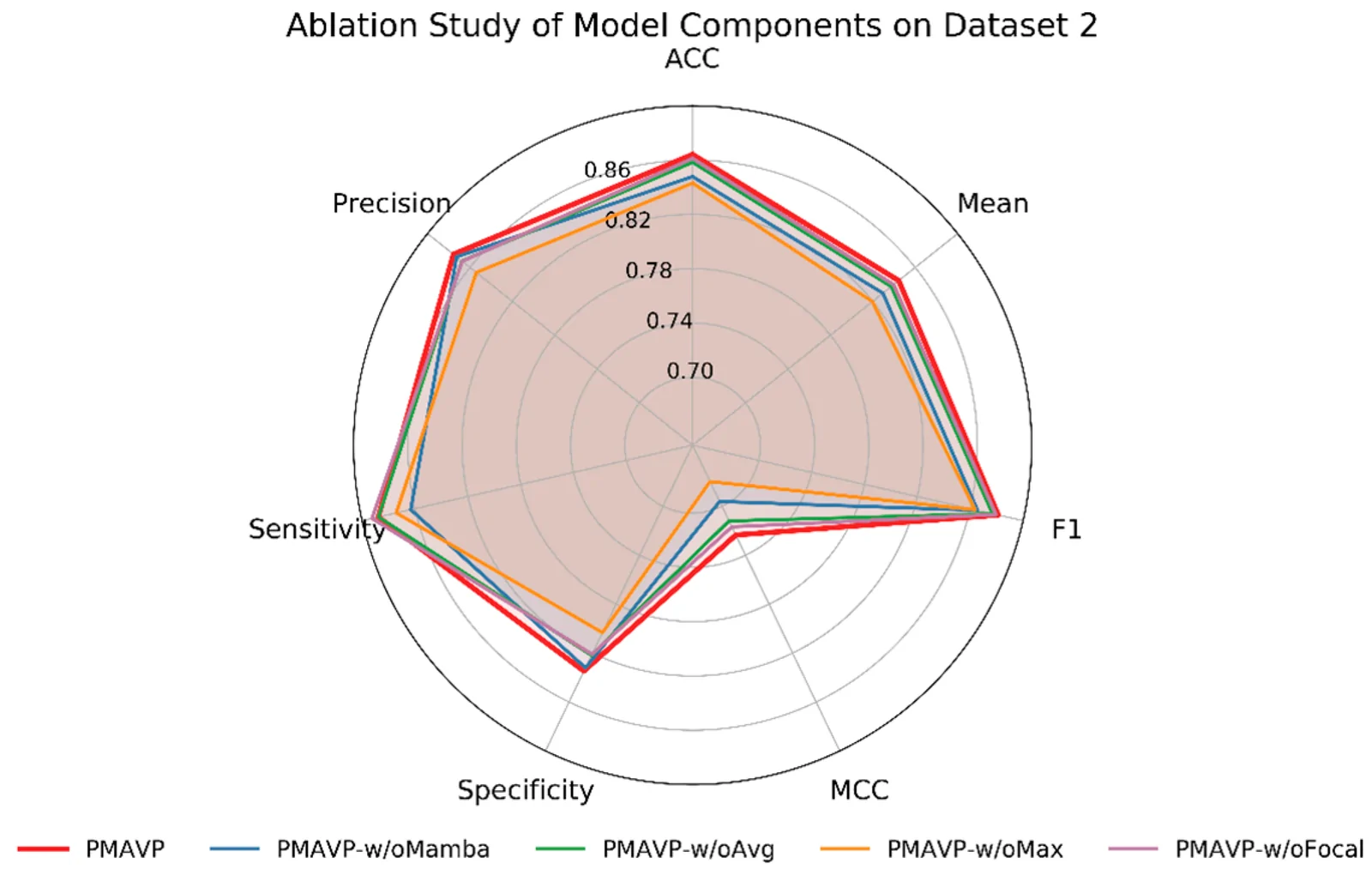
Radar chart of the ablation results for PMAVP and its model variants on Dataset 2.

Figure 1 presents the ablation results of PMAVP and its model variants. Overall, PMAVP achieves the best performance across all evaluation metrics, demonstrating the effectiveness of the proposed components and their complementary contributions. Among them, the Mamba module and the global max pooling branch contribute most significantly to performance improvement. Specifically, introducing the Mamba module leads to consistent improvements across multiple evaluation metrics, particularly MCC and F1 score, highlighting its critical role in enhancing discriminative capability. Although removing the global max pooling layer results in the most severe performance degradation, the removal of the Mamba module also causes substantial declines across nearly all metrics, indicating that Mamba serves as the core sequence modeling component of PMAVP. By effectively capturing long-range dependencies and contextual relationships within peptide sequences, the Mamba module provides robust global feature representations for downstream classification tasks.

In contrast, removing the global average pooling layer results in only a moderate decline in performance, whereas removing the global max pooling layer leads to the most substantial performance degradation, particularly in terms of MCC and F1 score, highlighting its critical role in extracting discriminative local features associated with antiviral activity. These findings suggest that the Mamba module and global max pooling play complementary roles in PMAVP, with Mamba primarily responsible for global sequence modeling and global max pooling focusing on local discriminative feature extraction. Furthermore, replacing Focal Loss with the standard cross-entropy loss also results in performance deterioration, suggesting that Focal Loss effectively alleviates the impact of class imbalance and improves the recognition of hard samples.

**Figure 2:**
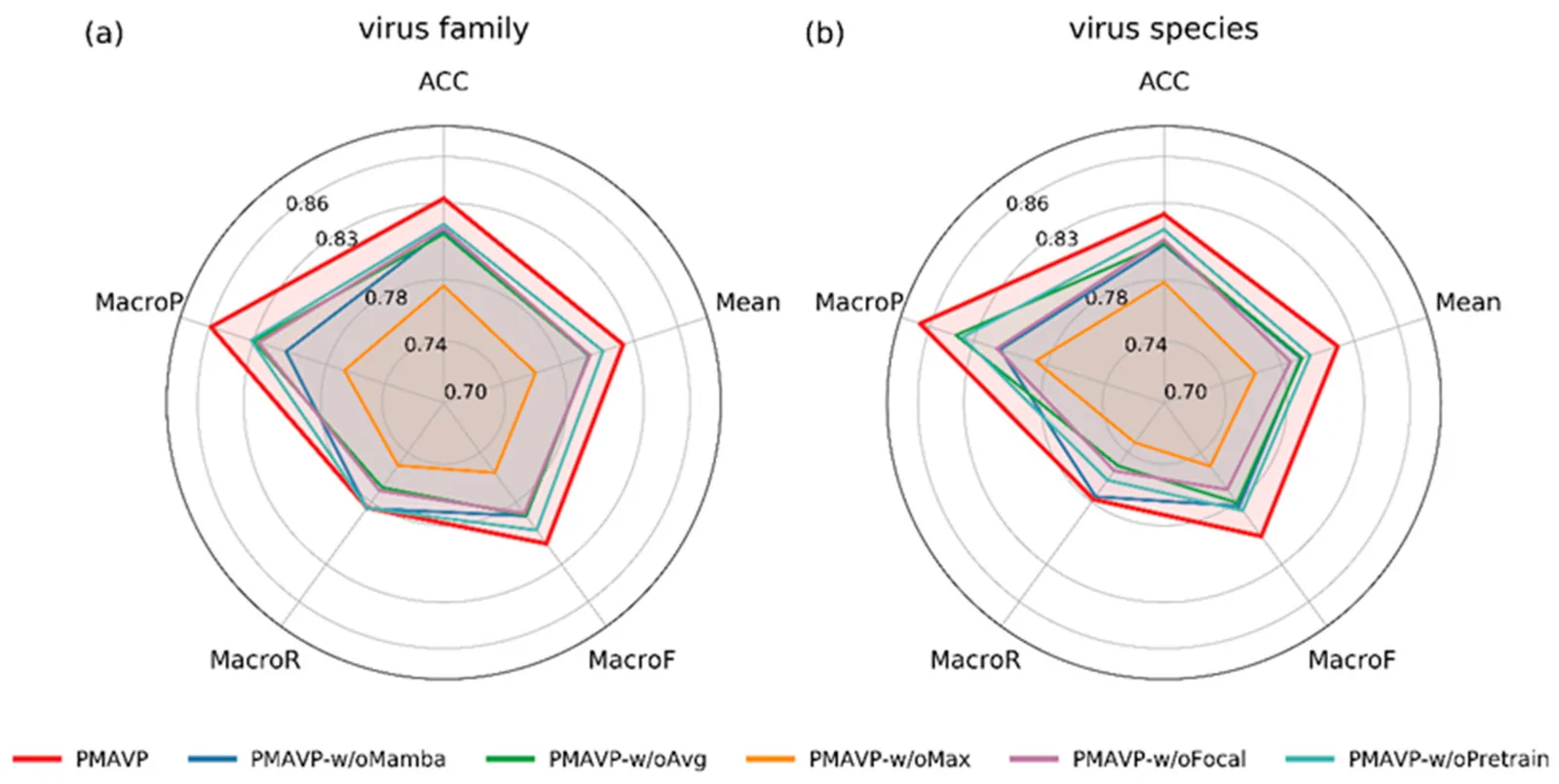
Radar charts of the ablation results for PMAVP and its model variants in the classification of (**a**) virus families and (**b**) species.

To further evaluate the contributions of the key components in AVP functional activity classification, ablation experiments were conducted based on the proposed PMAVP model. Five model variants were constructed by progressively removing or replacing core components, including the Mamba module, the dual-pooling structure (global average pooling and global max pooling), the Focal Loss function, and the pretraining strategy. These variants—namely, PMAVP-w/oMamba, PMAVP-w/oAvg, PMAVP-w/oMax, PMAVP-w/oFocal, and PMAVP-w/oPretrain—were designed to systematically analyze the impact of each component on model performance, thereby validating their effectiveness and necessity in functional activity classification tasks. The experimental results are presented in Figure 2.

PMAVP achieves the best performance across all evaluation metrics, demonstrating the effectiveness of the proposed architecture and the positive contributions of its key components. Moreover, the performance trends of different model variants remain generally consistent across functional activity prediction tasks, indicating good stability and generalization capability. Specifically, removing the Mamba module results in substantial performance degradation, particularly in MacroF and MacroP, suggesting that the module effectively captures complex sequence dependencies and enhances discrimination among functional categories. Removing the global average pooling layer causes only a moderate decline in performance, whereas removing the global max pooling layer leads to the most severe deterioration, indicating its critical role in extracting discriminative local features from peptide sequences. In addition, replacing Focal Loss with the standard cross-entropy loss slightly reduces performance, suggesting its effectiveness in alleviating class imbalance and improving hard sample recognition. Removing the pretraining strategy also causes noticeable degradation across multiple metrics, further demonstrating the importance of pretrained representations in enhancing feature representation and generalization performance.

Overall, the ablation results for both AVP classification and functional activity prediction exhibit highly consistent patterns. The Mamba module and global max pooling serve as the primary drivers of performance improvement, while global average pooling and Focal Loss provide complementary optimization effects. Meanwhile, the pretraining strategy further strengthens model capability in more complex functional activity prediction tasks. The effective collaboration among these components enables PMAVP to achieve strong performance in both AVP identification and functional activity prediction, thereby validating the rationality, stability, and generalization ability of the proposed architecture.

### 2.2. Performance Comparison with State-of-the-Art Methods for AVP Identification

**Figure 3:**
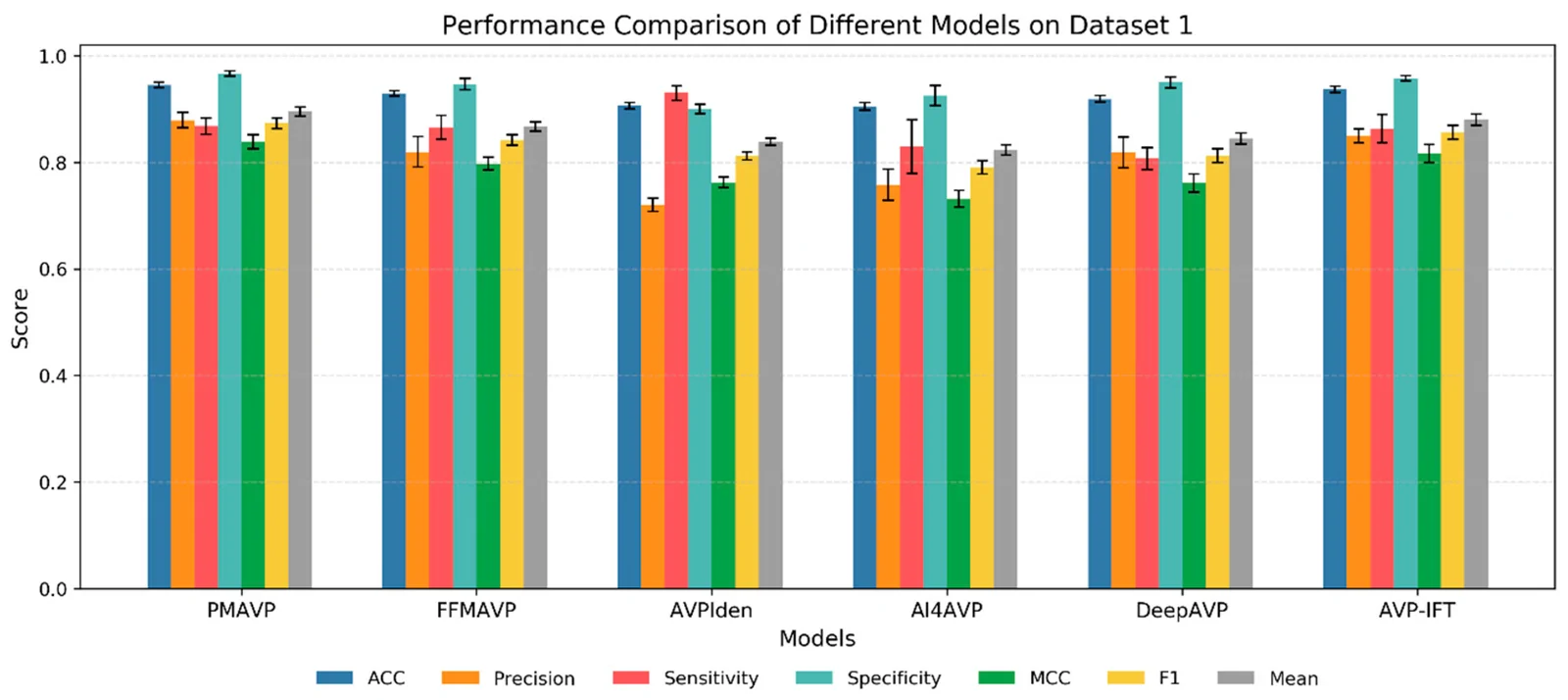
Comparison of AVP classification performance between PMAVP and existing methods on Dataset 1. The black error bars represent standard deviations. The same description also applies to Figure 4 and Figure 5.

To evaluate the performance of our model in AVP classification tasks, several representative state-of-the-art methods, including FFMAVP [15], AVPIden [10], AI4AVP [14], DeepAVP [11] and AVP-IFT [16], were selected for comparative analysis across three benchmark datasets. To ensure fairness, all datasets were randomly divided into training and independent test sets at a ratio of 4:1, followed by four-fold cross-validation to assess model robustness. For experimental settings, all compared models were trained under identical conditions with a batch size of 64 and 30 training epochs. The learning rate of PMAVP was set to 0.0001, while the remaining models were configured according to the optimal hyperparameters recommended in their original studies. To reduce performance fluctuations caused by random initialization, all evaluation metrics were averaged over five independent runs. PMAVP outperforms most competing methods on Dataset 1 and achieves the best or near-best performance across multiple key evaluation metrics, demonstrating strong overall classification capability (Figure 3). Specifically, PMAVP achieves the highest scores in ACC (0.946), precision (0.880), specificity (0.967), MCC (0.839), F1 score (0.874), and the overall mean metric (0.896), indicating clear advantages in overall classification accuracy, false-positive control, and comprehensive discriminative performance. Although the sensitivity of PMAVP is slightly lower than that of AVPIden, it remains at a high level, suggesting that the model achieves a favorable balance between reducing false negatives and controlling false positives. Notably, PMAVP shows significant improvements in MCC and F1 score, two important indicators of overall model performance, further demonstrating its robustness and reliability under class-imbalanced conditions.

**Figure 4:**
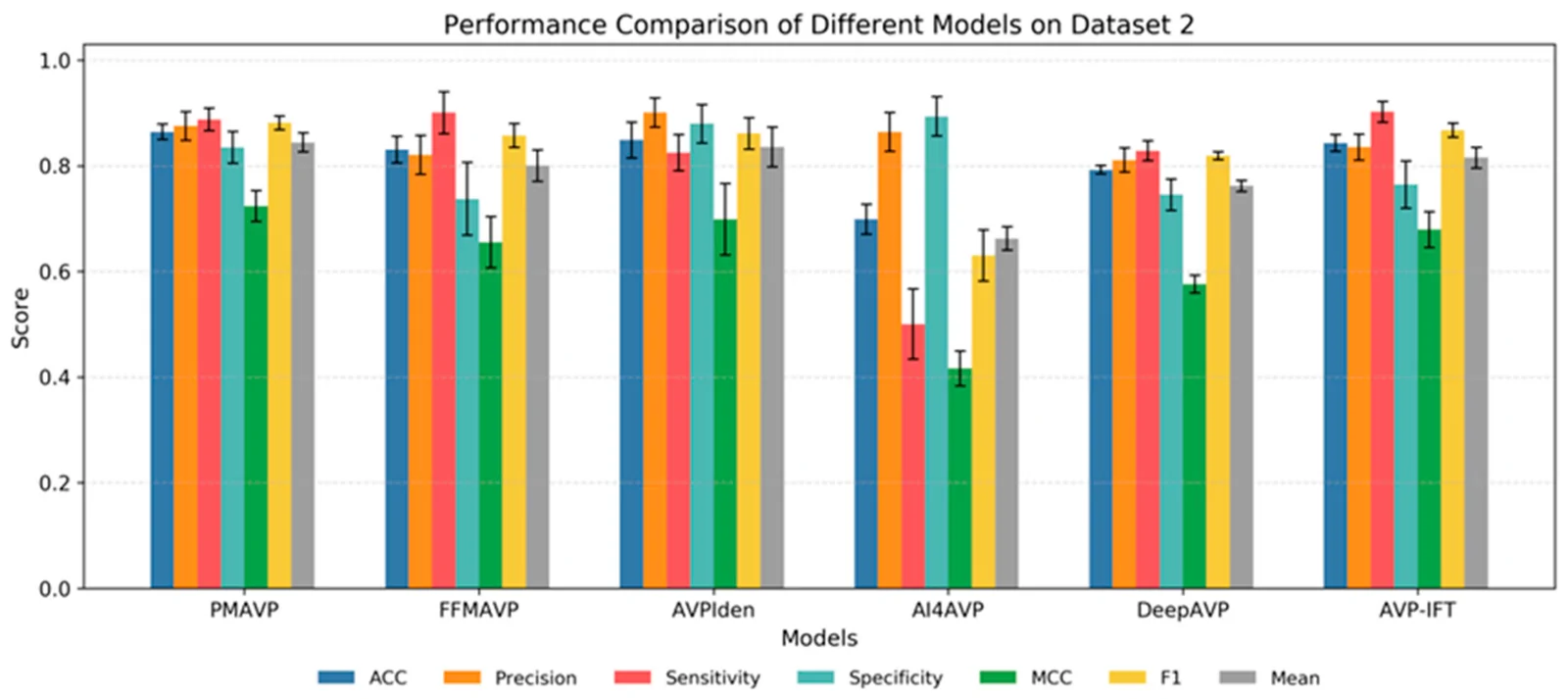
Comparison of AVP classification performance between PMAVP and existing methods on Dataset 2.

As shown in Figure 4, PMAVP demonstrates strong overall performance across multiple key evaluation metrics on Dataset 2. Specifically, PMAVP achieves the best results in ACC (0.864), MCC (0.723), F1 score (0.881), and the overall mean metric (0.844). In addition, PMAVP maintains a high sensitivity score (0.888), indicating strong capability in positive sample recognition and balanced overall discrimination. In comparison, although AI4AVP achieves slightly better performance in specificity (0.894), its MCC and F1 score are inferior to those of PMAVP, suggesting relatively weaker overall discriminative capability. Furthermore, AVP-IFT shows the highest sensitivity (0.902), but its performance in precision and MCC remains lower than that of PMAVP, reflecting certain limitations in false-positive control and overall classification performance.

**Figure 5:**
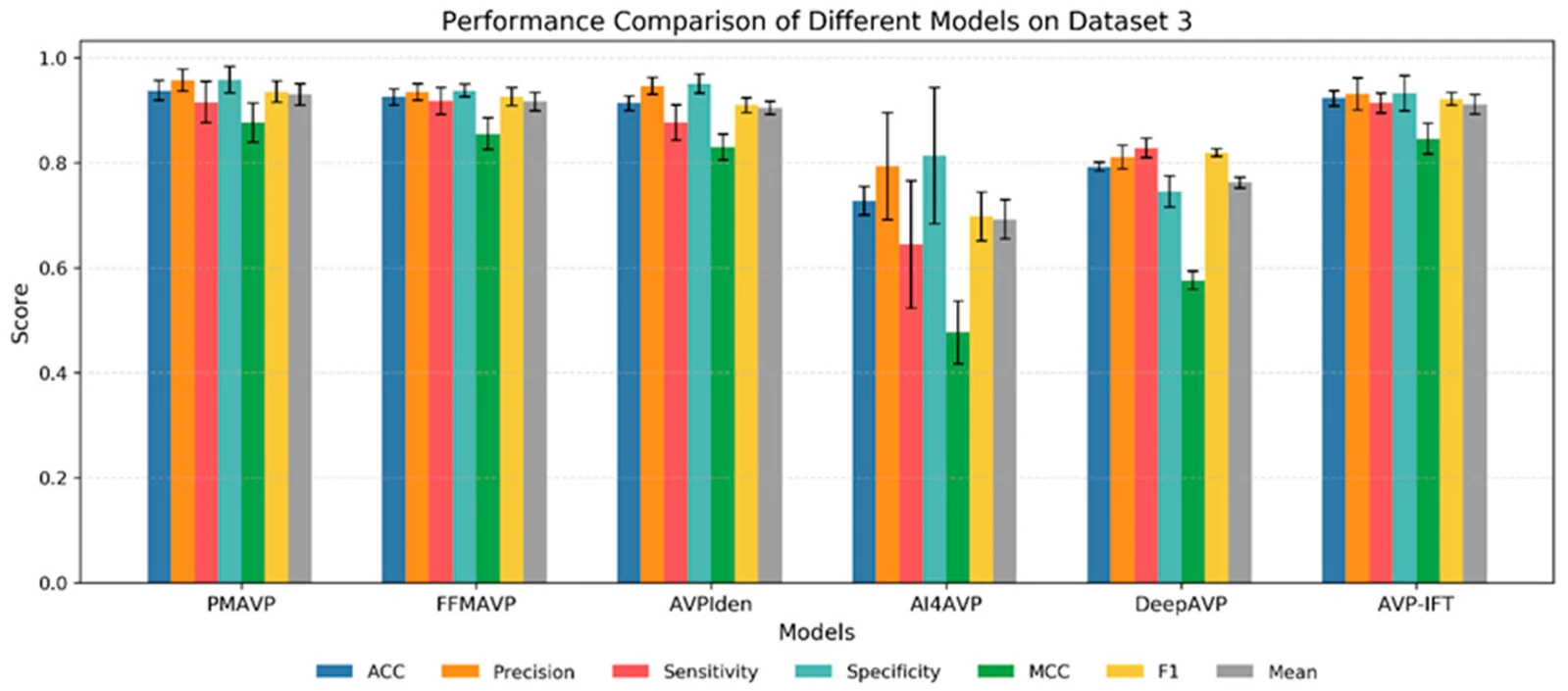
Comparison of AVP classification performance between PMAVP and existing methods on Dataset 3.

As depicted in Figure 5, PMAVP outperforms most competing methods on Dataset 3 and achieves the best or near-best results across multiple key evaluation metrics, demonstrating its strong overall discriminative capability. Specifically, PMAVP achieves the highest scores for ACC (0.938), precision (0.957), specificity (0.958), MCC (0.876), F1 score (0.936), and the overall mean metric (0.930), indicating clear advantages in overall classification accuracy, false-positive control, and discriminative performance under class-imbalanced conditions. PMAVP also maintains a high sensitivity score (0.916), indicating that the model achieves a favorable balance between positive and negative sample recognition. Although FFMAVP exhibits marginally better performance in sensitivity, its results on other metrics are less balanced, whereas AI4AVP and DeepAVP exhibit noticeably inferior overall performance. Notably, PMAVP consistently demonstrates relatively small standard deviations across all evaluation metrics on all three datasets, indicating strong stability and reproducibility over multiple independent experimental runs.

**Figure 6:**
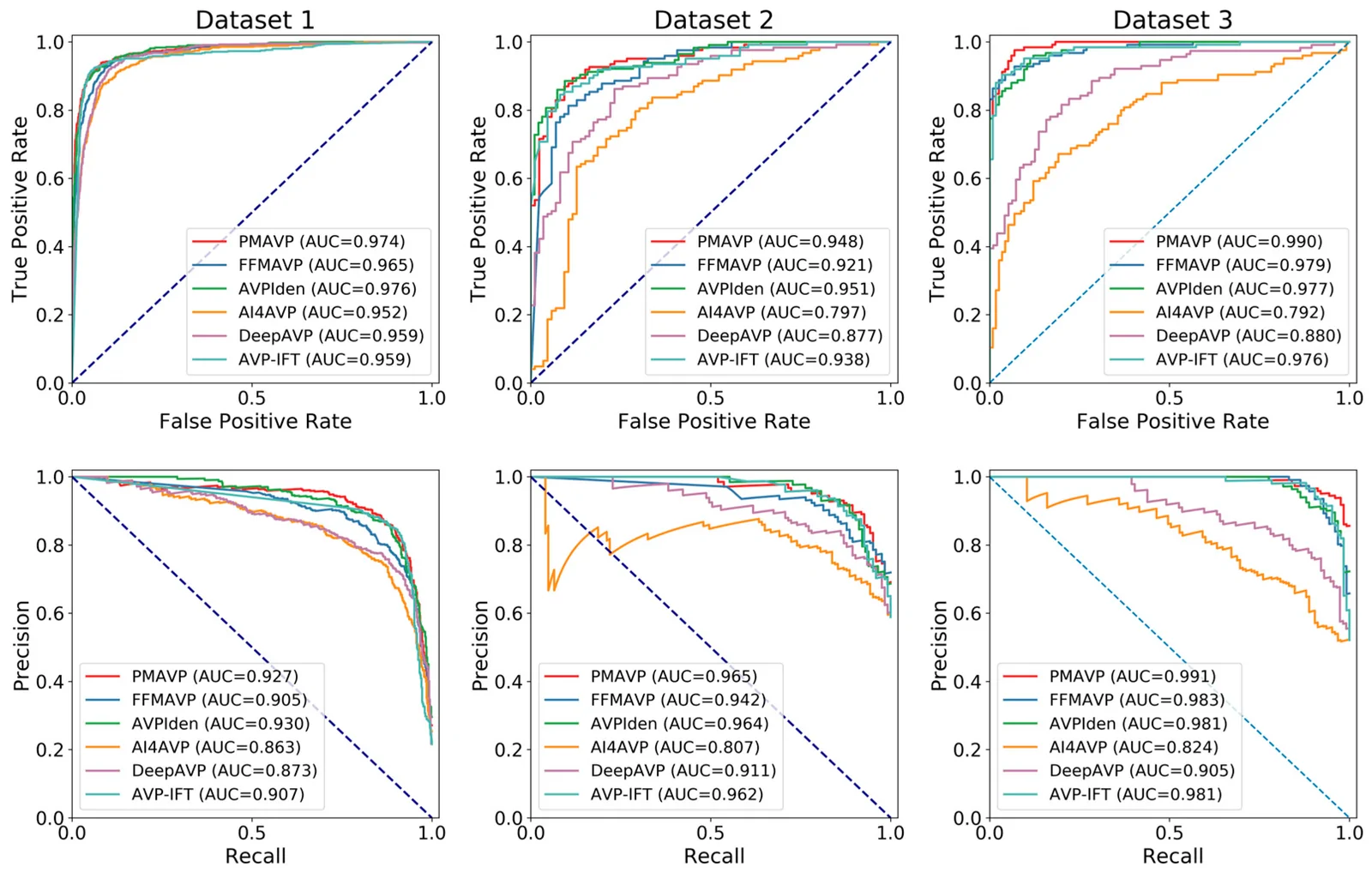
ROC curves (**top**) and precision–recall curves (**bottom**) of PMAVP and competing methods based on the best results obtained from five experimental runs across three datasets.

PMAVP also achieves superior or comparable performance in both receiver operating characteristic (ROC) curve and precision–recall (PR) curve analyses across all datasets (Figure 6). Its ROC curves are consistently closer to the upper-left corner, while the PR curves generally remain above those of competing methods, indicating stronger discriminative capability and better handling of class imbalance. Overall, PMAVP consistently achieves the best or near-best performance across the three benchmark datasets, demonstrating strong overall classification capability and robust generalization performance. PMAVP obtains superior results in key comprehensive metrics, including MCC, F1 score, and the overall mean, indicating its strong discriminative ability under class-imbalanced conditions. Although certain competing methods achieve slightly better performance on individual metrics such as sensitivity and specificity, their overall performance remains less balanced than that of PMAVP. A consistent trend across the three datasets is that PMAVP maintains a favorable balance between positive sample recognition and false-positive control, resulting in more stable and reliable classification performance. Moreover, the consistently small standard deviations observed across repeated experiments further demonstrate the robustness and reproducibility of the proposed model.

### 2.3. Performance Comparison with State-of-the-Art Methods for AVP Functional Prediction

We further applied PMAVP to AVP functional activity prediction tasks and compared its performance with that of several mainstream methods, including FFMAVP [15] and AVPIden [10]. Systematic evaluations were conducted at both the virus family and virus species levels to demonstrate the advantages of the proposed model in multi-class classification tasks. To ensure fairness, all models were trained under identical experimental conditions, with the number of training epochs and batch size fixed at 20 and 64, respectively. During the pretraining stage, the epoch number and batch size were set to 15 and 64, respectively, and the learning rate was fixed at 0.0001 for both the pretraining and fine-tuning stages. The remaining competing methods were configured according to the optimal learning rates recommended in their original studies to ensure that each model operated under its best-performing settings. The average experimental results are summarized in Table 2.

PMAVP demonstrates strong overall performance in AVP functional activity classification tasks. At the virus family level, PMAVP achieves the best results in several key metrics, including macro-precision (0.859), macro-F1 score (0.813), and the overall mean metric (0.823), indicating clear advantages in overall classification performance and inter-class balance. ur model also exhibits comparable performance relative to FFMAVP in terms of accuracy (0.833). Although its macro-recall is slightly lower than that of FFMAVP, the difference is marginal, suggesting that PMAVP maintains strong recall capability while achieving higher precision. At the virus species level, the performance differences among the compared models are relatively smaller; however, PMAVP still demonstrates strong competitiveness. Specifically, PMAVP achieves the highest macro-precision (0.867), indicating superior accuracy in positive class prediction for multi-class tasks. In addition, its accuracy (0.823) and macro-F1 score (0.808) are close to the best-performing results, reflecting stable overall performance. By comparison, FFMAVP achieves slightly better results in accuracy and macro-F1 score, whereas AVPIden achieves higher macro-recall but performs relatively weaker on other metrics, suggesting a less balanced classification capability. Furthermore, PMAVP exhibits consistently small standard deviations across all evaluation metrics, demonstrating strong stability and reliability over multiple cross-validation runs. Overall, PMAVP shows strong comprehensive performance in both virus family and species classification tasks, with particularly notable advantages in macro-precision and macro-F1 score, further validating its effectiveness and generalization capability in complex functional classification scenarios.

### 2.4. Visualization Analysis of PMAVP for AVP Classification

**Figure 7:**
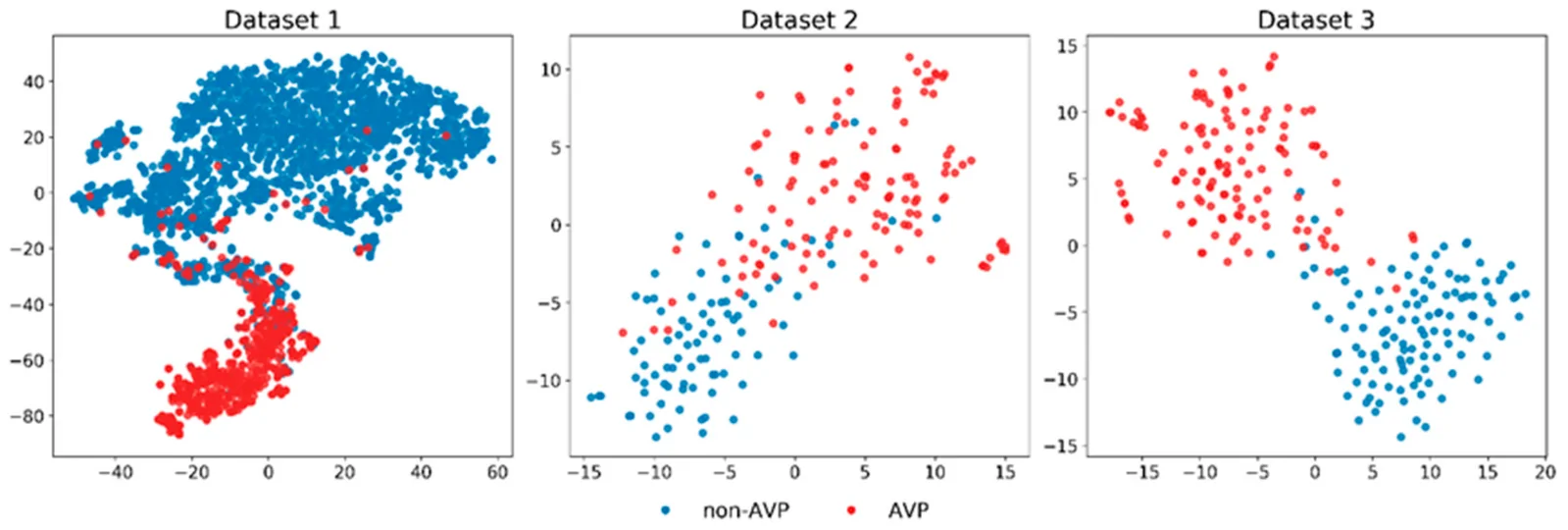
t-SNE visualization of feature distributions generated by PMAVP on three datasets.

To further investigate the feature learning and class discrimination capabilities of PMAVP in AVP classification tasks, t-distributed stochastic neighbor embedding (t-SNE) [22] was employed to visualize the output features of the fully connected layer (units = 256) on the test sets. By projecting samples from different classes into a two-dimensional space, the distribution structure and discriminative capability of the learned feature representations can be intuitively illustrated. The feature distributions of PMAVP on the three datasets are presented in Figure 7, where red and blue points represent AVP and non-AVP samples, respectively. Overall, the learned feature representations exhibit clear class separation trends across all datasets, indicating that PMAVP effectively extracts discriminative features. Although some overlap between the two classes can still be observed in local regions on Dataset 1, the overall distribution boundary remains relatively distinct, suggesting that the model achieves preliminary class separation under complex data distributions. The separation boundary between AVPs and non-AVPs becomes more evident on Dataset 2, with the two classes exhibiting clearer clustering patterns in the feature space, indicating stronger discriminative capability. The two classes are almost completely separated on Dataset 3, with the clearest class boundaries among the three datasets, demonstrating the strong feature representation and classification capability of PMAVP on relatively balanced data. These findings further suggest that PMAVP can learn stable and highly discriminative feature representations when handling balanced datasets.

### 2.5. Model Interpretation

**Figure 8:**
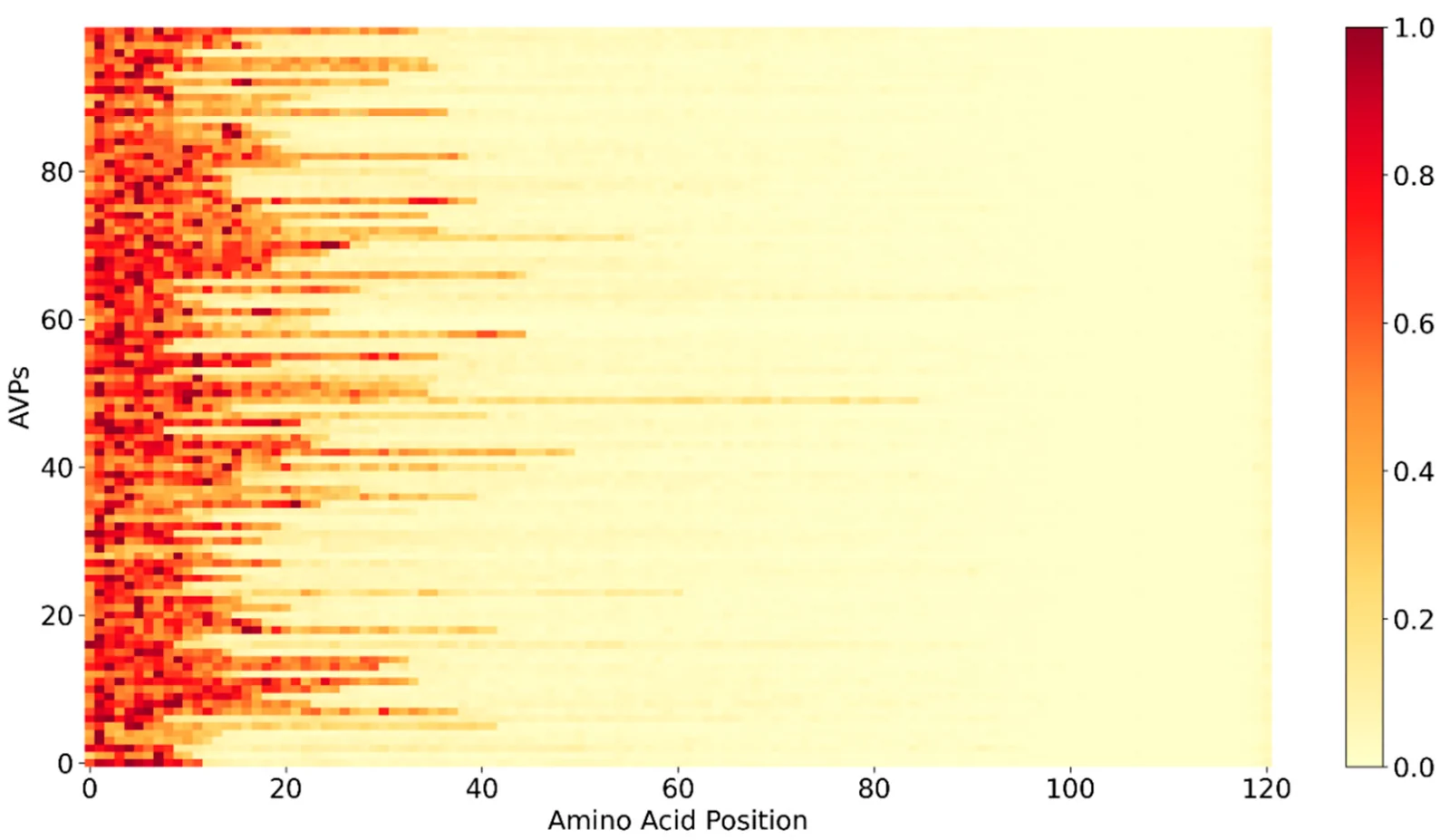
Heatmap of amino acid positional contribution analysis for PMAVP based on DeepSHAP. The color intensity (from light yellow to dark red) represents the normalized contribution scores of residues.

To further investigate the internal decision-making mechanism of PMAVP in AVP recognition tasks, DeepSHAP [21] was employed to perform an interpretability analysis of the proposed model. Specifically, 100 AVP sequences were randomly selected from Dataset 1 as target samples, while the complete test set was used as the background reference for baseline evaluation. By normalizing the contribution scores generated by DeepSHAP, the amino acid positional contribution heatmap shown in Figure 8 was obtained. The heatmap results show that the high-contribution regions, indicated by the dark-red areas, were mainly concentrated at the N-terminus of the sequences, particularly within the first 30–40 residues of AVPs. Previous studies have suggested that the N-terminal regions of some membrane-active AVPs exhibit pronounced amphipathic properties. Hydrophobic aromatic residues, such as tryptophan and phenylalanine, may facilitate the initial adsorption and subsequent insertion of peptides into the phospholipid bilayer of the viral envelope, thereby disrupting membrane stability and inhibiting viral infection [23]. The strong focus of PMAVP on this functional region suggests that the model successfully captures the key features associated with antiviral activity. In addition, the heatmap demonstrates the adaptive capability of PMAVP in handling variable-length sequences. As the sequence position exceeds approximately 40 residues, the contribution scores gradually decrease and become more uniform. Since AVP sequences vary considerably in length, the model can adaptively identify informative regions while assigning very low contribution weights to padding tokens or non-critical residues located near the sequence termini. Notably, the model exhibits highly consistent response patterns across different sequences. Although the amino acid compositions of the AVPs differ substantially, strong feature activations are consistently observed within the first 40 positions. This cross-sequence consistency indicates that PMAVP does not rely on sample-specific characteristics for classification, instead learning common structural patterns and physicochemical property distributions shared among AVPs, thereby enhancing its generalization capability and stability.

Our ablation study results also demonstrate that the Mamba module and global max pooling layer are the most significant contributors to performance improvement. Specifically, the Mamba module effectively models long-range dependencies within peptide sequences, enabling the model to capture contextual information associated with the key functional regions in the N-terminus. Meanwhile, the global max pooling layer enhances the recognition of critical motifs by emphasizing highly responsive local features. The complementary interplay between these two components allows PMAVP to preserve global sequence modeling capability while simultaneously highlighting discriminative local regions, thereby significantly improving feature representation and classification performance.

## 3. Discussion

AVPs have attracted increasing attention for combating emerging infectious diseases and drug resistance associated with conventional antiviral agents [7]. However, the traditional discovery and development of AVPs largely rely on extensive experimental screening, which is often time-consuming and costly [24]. In this context, computational approaches have provided an efficient alternative for AVP research. By analyzing and screening large-scale peptide sequences, computational models enable researchers to rapidly identify potential antiviral candidates and facilitate subsequent sequence optimization and functional validation, thereby reducing experimental costs and improving research efficiency. In recent years, the rapid development of deep learning techniques has led to their increasing application in AVP prediction tasks. Deep learning models can extract informative patterns associated with antiviral activity from large numbers of candidate sequences, thereby improving the efficiency of active peptide screening. Nevertheless, several limitations remain in current studies. On the one hand, class imbalance is a common issue in AVP datasets, which may negatively affect model training and prediction performance. On the other hand, many deep learning models still rely heavily on manually engineered features, which are often time-consuming to construct and may not be sufficiently reliable. In some cases, these handcrafted features may even introduce irrelevant information and interfere with the model’s learning process. Moreover, existing methods often lack sufficient interpretability, making it difficult to associate prediction results with clear biological mechanisms. Although considerable progress has been made in the design of model architectures, there is still room for improvement in handling complex sequence relationships and identifying key patterns closely associated with biological functions.

To address these pressing challenges, we propose PMAVP, a novel deep learning framework that integrates the ProtT5 pretrained protein language model with a state-of-the-art Mamba-inspired sequence modeling architecture. The proposed model can extract rich, context-aware semantic representations from large-scale peptide sequences while efficiently modeling long-range dependencies within sequences. Notably, the ProtT5 model embeddings used in this study have been shown to encode physicochemical properties in their latent space. Therefore, we did not perform physicochemical feature engineering during model development. However, we acknowledge that post hoc physicochemical profiling of the dataset could provide additional insights into data diversity; we leave this for future work. We employ a dual-pooling strategy that combines global average pooling and global max pooling to jointly capture global contextual information and discriminative local functional features for effective feature aggregation. To mitigate the class imbalance problem prevalent in AVP datasets, we introduce the Focal Loss function, which directs the model to focus more on hard-to-classify samples and thereby improves overall classification performance.

For downstream functional activity prediction tasks, we further incorporate a transfer learning paradigm. Specifically, the model is first pretrained on the AVP binary classification task to learn discriminative and generalizable sequence representations. The pretrained model weights are then transferred to downstream functional activity classification tasks and further fine-tuned on the corresponding functional activity datasets, enabling the model to gradually adapt to the more complex multi-class distributions of these tasks. Ablation studies confirm that the Mamba module and global max pooling layer are the primary contributors to the model’s superior performance. Experimental results show that PMAVP achieves satisfactory performance in terms of prediction accuracy, stability and computational efficiency. Moreover, DeepSHAP-based interpretability analysis reveals that the first 40 amino acid positions are critical for AVP prediction, providing valuable insights into the key functional regions associated with antiviral activity.

While PMAVP achieves promising results in both AVP identification and functional activity prediction, several important directions remain for further investigation. First, the size of currently available AVP datasets remains relatively small, which limits the performance and generalization ability of deep learning models. In future work, generative adversarial networks (GANs) or diffusion models could be used to generate biologically plausible AVP sequences, thereby expanding the training dataset, increasing data diversity, and enhancing model robustness. Second, our interpretability analysis indicates that the first 30–40 residues of AVP sequences contribute most significantly to model prediction. However, these findings are currently based solely on computational inference and require experimental validation. Future work will experimentally validate these computational findings to elucidate the precise functional roles of these critical regions. Future truncation or swap experiments are also required to dissect the relative contributions of length versus sequence-specific effects. Finally, the Mamba module implemented in our model is a simplified version inspired by the core principles of the original Mamba architecture. Future research will focus on developing a more optimized Mamba framework specifically tailored for peptide sequence modeling, including low-level CUDA optimizations and specialized state-space formulations. Recent advances in equivariant neural networks that respect rotational and translational symmetries offer a mathematically principled way to learn from 3D coordinates. We envision that as predicted structural databases expand, such architectures could be incorporated into our Mamba-inspired module to enhance both prediction robustness and the biological plausibility of the identified features. Additionally, future work should integrate molecular docking or AlphaFold-Multimer predictions for top candidates to provide structural hypotheses for experimental testing. Such analyses will be valuable for candidates predicted to act via direct protein–protein inhibition mechanisms.

## 4. Materials and Methods

### 4.1. Dataset Collection and Preparation

We used three benchmark datasets—namely, Dataset 1, Dataset 2 and Dataset 3—to evaluate the effectiveness of our model. Dataset 1 was collected and processed by Cao et al. [15], with the original sequences obtained from public databases including AVPdb [25], HIPdb [26] and UniProt [27]. Dataset 1 contains 2762 AVPs and 10,089 non-AVPs, with sequence lengths ranging from 7 to 120 amino acids. Dataset 2 was processed by Guan et al. [16], constructed through redundancy removal and data cleaning based on 604 experimentally validated AVPs and 452 validated non-AVPs. After filtering sequences containing invalid amino acid residues, the final dataset consisted of 600 AVPs and 444 non-AVPs, with sequence lengths ranging from 6 to 107 amino acids. Dataset 3 was also constructed by Guan et al. [16], containing 600 experimentally validated AVPs and 599 non-AVPs, with peptide lengths ranging from 6 to 107 amino acids. Standard binary labels were assigned, with AVPs and non-AVPs were denoted as 1 and 0, respectively. More detailed information can be found in Table 3.

**Figure 9:**
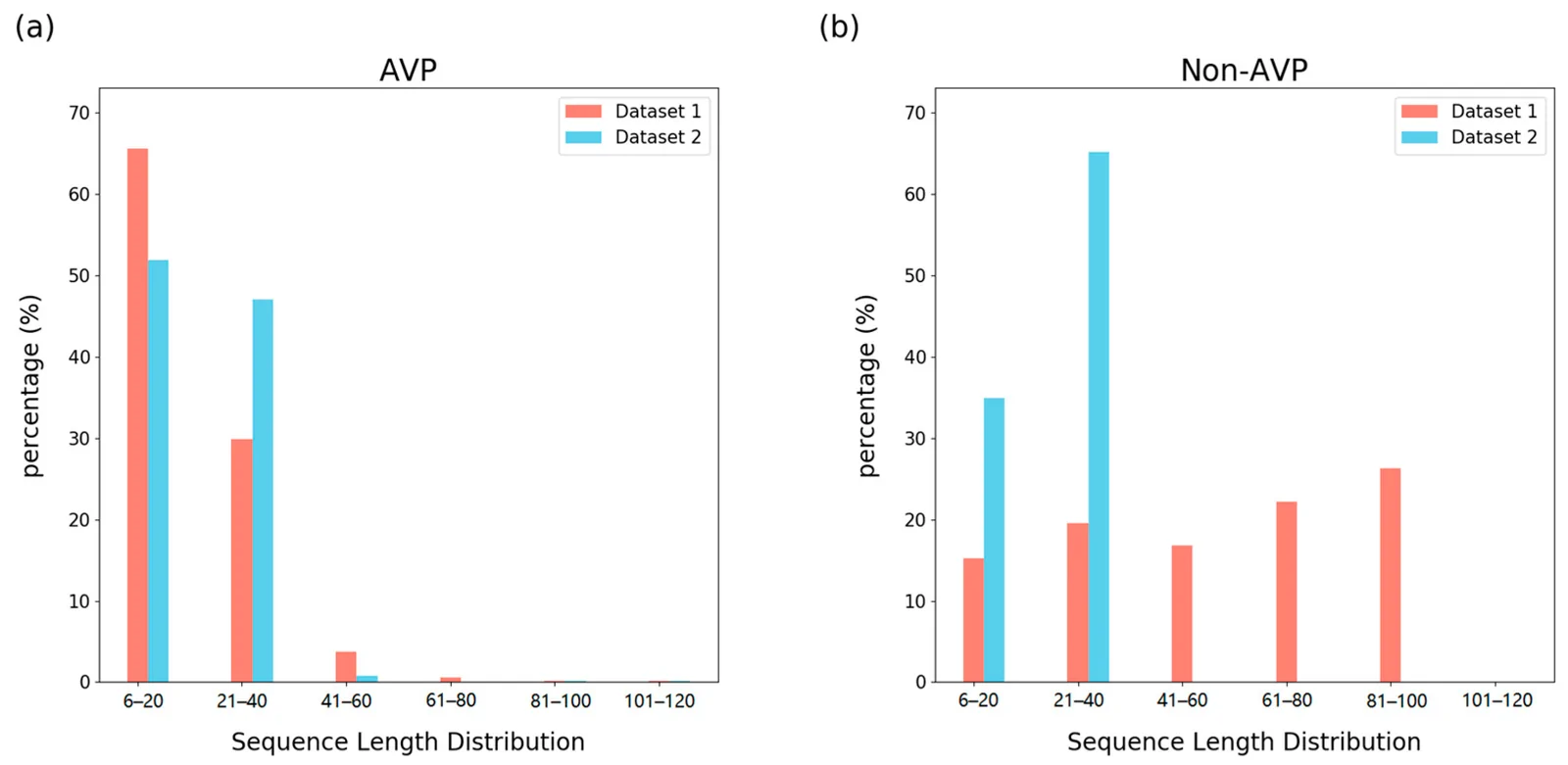
Histograms showing the sequence-length distributions of (**a**) AVPs and (**b**) non-AVPs on Dataset 1 and Dataset 2.

Figure 9 illustrates the sequence-length distributions of AVPs and non-AVPs on Dataset 1 and Dataset 2. Overall, AVPs in both datasets are predominantly concentrated within shorter length ranges. Specifically, approximately 95.33% of AVPs in Dataset 1 contain, at most, 40 amino acids, while this proportion increases to 98.83% on Dataset 2. This observation confirms that AVPs generally exist as short peptides, consistent with their biological role as natural defense molecules that exert functions through relatively compact sequences [28]. In contrast, the length distributions of non-AVPs differ substantially between the two datasets. Non-AVPs exhibit a relatively dispersed distribution across length intervals and are biased toward longer sequences on Dataset 1, with the 81–100 amino acid interval accounting for the highest proportion (26.25%). However, non-AVPs are mainly concentrated within the 21–40 amino acid range, accounting for approximately 65.09% of samples on Dataset 2. These results indicate that the overlap in sequence-length distribution between AVPs and non-AVPs is substantially greater on Dataset 2, making the classification task more challenging.

We adopted the AVP functional activity dataset constructed by Cao et al. [15] for functional activity prediction. Specifically, AVPs were categorized into six virus families—namely, Coronaviridae, Flaviviridae, Herpesviridae, Orthomyxoviridae, Paramyxoviridae and Retroviridae, containing 224, 469, 262, 109, 268, and 996 samples, respectively. Additionally, these AVPs were grouped into eight target virus species, including feline immunodeficiency virus (FIV), hepatitis C virus (HCV), human immunodeficiency virus (HIV), human parainfluenza virus type 3 (HPIV3), herpes simplex virus type 1 (HSV1), influenza A virus (INFVA), respiratory syncytial virus (RSV), and SARS-CoV, with 101, 431, 873, 87, 210, 108, 118, and 188 samples, respectively. Note that only a small number of AVPs simultaneously appeared across multiple target virus families or species, and duplicate sequences belonging to two or more categories were removed to ensure better independence among classes. During data preprocessing, at least 100 sequences were retained for each virus family, and at least 80 sequences were preserved for each virus species. For subsequent model training and classification tasks, the six virus families were encoded as labels 0 to 5, while the eight virus species were encoded as labels 0 to 7.

### 4.2. PMAVP Model Architecture

**Figure 10:**
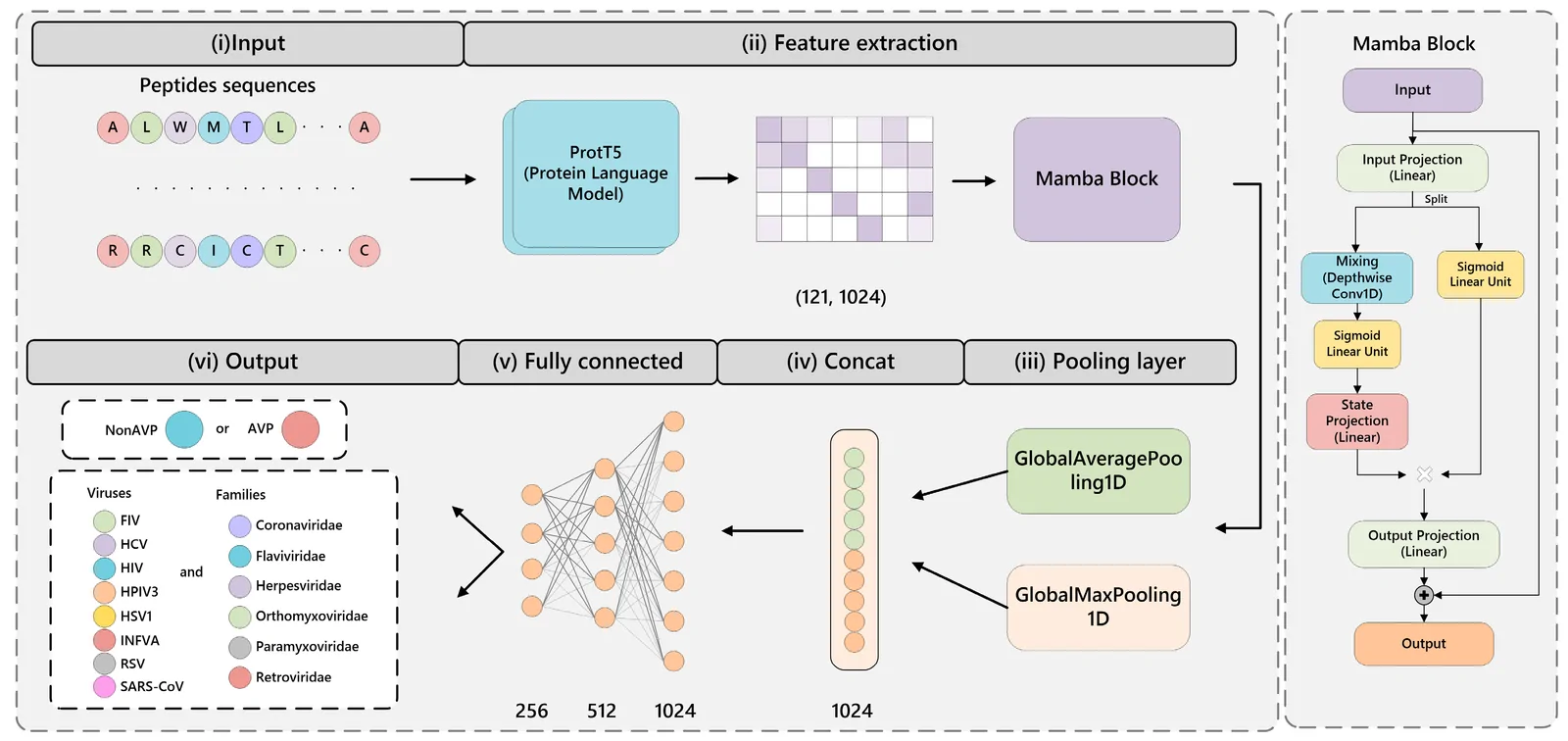
Architecture of PMAVP. Our model consists of six components: (**i**) input and (**ii**) feature extraction, for which each peptide sequence is encoded to obtain deep representations; (**iii**) the pooling layer, which employs a dual-pooling strategy to extract both average and salient response features; (**iv**) the concatenation layer, which is applied for feature fusion; (**v**) the fully connected layers, with dimensions of 1024, 512 and 256; (**vi**) and the output layer, which identifies whether a given sequence is an AVP, as well as its functional activity.

Figure 10 depicts the architecture of our model. It comprises six components—namely, the input layer, feature extraction module, pooling layer, concatenation layer, fully connected layers and the output layer. Initially, peptide sequences are encoded using the ProtT5 pre-trained protein language model, which maps discrete amino acid sequences into high-dimensional continuous representations. The resulting feature matrices are then fed into a Mamba-inspired module for deep sequence modeling. Notably, Mamba can leverage a selective state-space model to capture long-range dependencies with significantly reduced computational cost relative to traditional self-attention mechanisms. We apply global average pooling and global max pooling to aggregate sequence-level information. The former captures global contextual features, while the latter emphasizes salient local patterns associated with functional motifs. The pooled features are concatenated and passed through a multi-layer fully connected network with dimensions of 1024, 512 and 256. Our model is designed to perform two tasks—namely, binary classification to identify whether a given sequence is an AVP and its functional activity across six virus families and eight virus species. The optimal hyperparameter settings of PMAVP are provided in Table 4.

### 4.3. ProtT5-Based Sequence Representation

ProtT5 [18] is a pretrained protein language model built upon the Transformer text-to-text transfer architecture [29] and trained on the large-scale BFD protein database [30]. It treats protein sequences as a form of biological language and leverages an encoder–decoder framework to model sequence semantics. During model pre-training, ProtT5 uses a sequence reconstruction objective analogous to a translation task, learning to recover corrupted or masked residues. This process enables the model to capture rich contextual and biochemical information embedded in protein sequences. As the T5 architecture can model long sequences and complex dependencies, ProtT5 generates high-quality embeddings that effectively characterize residue-level biochemical properties. Thus, ProtT5 is widely applied for extraction of informative sequence representations in protein function prediction tasks, providing robust features for downstream classification models.

### 4.4. Gated Conv-MLP Block, Inspired by Mamba’s Gating Mechanism

In recent years, the Mamba [19] module has emerged as an efficient sequence modeling framework. By introducing a selective state-space mechanism, Mamba enables dynamic feature updates while effectively capturing long-range dependencies in sequential data. Compared with Transformer-based architectures relying on self-attention, Mamba offers significant advantages in terms of computational complexity and memory efficiency. Mamba projects input sequences into a high-dimensional feature space and processes them recursively through state-space equations. By leveraging input-dependent dynamic parameters, the model selectively updates its internal states, allowing it to retain informative signals while suppressing irrelevant information. This mechanism enables adaptive control of information flow across time steps, thereby achieving a balance between global dependency modeling and local feature representation. Notably, the Mamba architecture relies on specialized scanning operators and low-level CUDA optimizations, making it difficult to implement within standard deep learning frameworks. Therefore, we design a simplified Mamba-inspired module that preserves its core principles. Specifically, the input sequence is projected into a higher-dimensional space via a fully connected layer, then split into two branches: a feature branch and a gating branch. The gating branch employs the Sigmoid Linear Unit (SiLU) activation function [31] to generate dynamic weights for selective information updating, thereby mimicking the selective update mechanism in Mamba. The formulation is given as follows:

(1) $$SiLU\left(x\right)=x\cdot \sigma (x)$$

where σ(x) denotes the Sigmoid function, i.e.,

(2) $$\sigma \left(x\right)=\frac{1}{1+e^{-x}}$$

This activation function introduces nonlinear modulation while preserving linear information, thereby enhancing the expressive power and smoothness of the model. To mimic the modeling of local dependencies in Mamba, a 1D convolutional neural network (1D-CNN) is applied to the feature branch. This operation performs channel-wise modeling, which helps capture local contextual information while maintaining low computational cost. In the original Mamba architecture, state updates are governed by continuous-time state-space equations. In this study, this process is approximated using two nonlinear, fully connected layers, with the Swish activation function [32] employed to enhance nonlinear representation capability. The formulation is given as follows:

(3) $$Swish\left(x\right)=x\cdot \sigma (x)$$

Compared with the ReLU activation, Swish is a smooth, non-monotonic, self-gated function. While ReLU completely suppresses negative inputs, Swish allows a small portion of negative values to pass through and provides smoother transitions around zero, thereby improving representational capacity. The features transformed by the state-space approximation are combined with the gating branch via element-wise multiplication. This operation enables selective feature filtering, preserving informative positions in the sequence while suppressing irrelevant signals. Residual connections are incorporated to alleviate the vanishing gradient problem in deep networks. In addition, layer normalization and dropout are applied to improve training stability and generalization performance. Overall, the proposed Mamba module is designed based on the core principles of the original Mamba architecture, including selective information flow control and local sequence mixing, while approximating state-space updates using one-dimensional convolutional layers and fully connected layers.

### 4.5. Feature Extraction

During the feature extraction stage, input peptide sequences are first fed into the ProtT5 pre-trained protein language model to obtain contextualized representations. Trained on large-scale protein sequence data, ProtT5 effectively captures contextual dependencies and latent semantic information within amino acid sequences, generating high-dimensional embeddings with rich biological significance. Given that the maximum sequence length in the dataset is 121, sequences shorter than this length are zero-padded to ensure consistent input dimensions. After encoding, each peptide is represented as a feature matrix of size 121 × 1024, where 121 denotes the sequence length and 1024 corresponds to the embedding dimension at each residue position. The high-dimensional features are linearly projected through a fully connected layer with 1024 hidden units before being fed into the Mamba module. This step reduces computational complexity and improves modeling efficiency by reorganizing and compressing the feature distribution while preserving essential semantic information. The projected features are then passed into the Mamba module for deep sequence modeling. By integrating gating mechanisms with local sequence-mixing operations, the model jointly captures long-range dependencies and local contextual patterns, thereby extracting more discriminative high-level representations. Compared with conventional self-attention-based approaches, this module achieves comparable modeling capability with improved computational efficiency. The hyperparameter settings of the Mamba module are detailed in Table 5. Finally, the output of the module remains a feature matrix of size 121 × 1024, which serves as a high-quality input for subsequent feature fusion and classification tasks.

### 4.6. Pooling and Feature Fusion

To further aggregate global sequence-level information, the output of the Mamba module is processed via both global average pooling and global max pooling, enabling feature compression from complementary perspectives. Global average pooling computes the mean across the sequence dimension, capturing the overall distributional characteristics and global semantic information of peptide sequences. This operation enhances robustness to input variations and enables the model to stably represent macroscopic biochemical properties. In contrast, global max pooling focuses on the most salient responses within the sequence by selecting the maximum activation value in each channel, thereby highlighting critical local patterns that are essential for classification. In the context of AVP identification, antiviral activity is often determined by specific functional motifs or key amino acid residues; thus, global max pooling is particularly effective at capturing these discriminative local signals. The features obtained from global average pooling and global max pooling are then concatenated along the channel dimension, integrating global contextual information with key local features to form a more comprehensive sequence representation. This dual-pooling strategy not only reduces the number of model parameters to some extent and alleviates overfitting but also enhances the model’s ability to capture the multi-scale characteristics of peptide sequences through multi-view feature fusion. The fused feature vector is subsequently fed into a fully connected classifier, where nonlinear transformations are applied to refine the features and learn discriminative representations. Specifically, the classifier consists of three fully connected layers with 1024, 512, and 256 neurons. Finally, the model outputs predictions for AVP classification and functional activity prediction.

### 4.7. Imbalanced Learning Strategy

We note that data imbalance is a serious issue on both Dataset 1 and Dataset 2. The number of non-AVPs in Dataset 1 reaches 10,089, whereas the number of AVPs is only 2762, resulting in a class ratio of approximately 1:3.65. Such a substantial imbalance can easily lead to majority-class bias during deep learning training, with model attaining high overall accuracy by favoring the majority class but performing poorly in identifying the minority class due to insufficient feature learning. Although Dataset 2 exhibits a relatively milder imbalance (1.35:1), its limited sample size (1044 sequences) makes the model more sensitive to any class-distribution skew, which may still adversely affect robustness. We employ the Focal Loss function [20] to mitigate the performance degradation caused by class imbalance. Focal Loss introduces a modulating factor that down-weights easy-to-classify samples while increasing the contribution of misclassified (hard) samples. This mechanism encourages the model to focus on challenging AVP samples rather than being dominated by the majority class during optimization. By emphasizing hard examples, PMAVP achieves improved discrimination of minority-class sequences while maintaining strong generalization performance. The formulation of Focal Loss is given as follows:

(4) $$L_{focal}=-\alpha_{t}{\left(1-p_{t}\right)}^{\gamma }\log\left(p_{t}\right),$$

where $p_{t}$ denotes the predicted probability of the true class, $\alpha_{t}$ is a balancing factor for class weights, and γ is the focusing parameter that controls the degree of emphasis on hard samples. We set $\alpha_{t}$ to 1 and γ to 0.75 for AVP identification and prediction. Furthermore, we set γ with a value of 2 and $\alpha_{t}$ with values of [0.16, 0.079, 0.14, 0.34, 0.14, 0.04, 0.01] and [0.17, 0.04, 0.02, 0.2, 0.08, 0.164, 0.1, 0.08, 0.01] for virus family and species prediction, respectively.

### 4.8. Pretraining and Implementation Details

For the AVP functional activity prediction task, the proposed PMAVP model is first pretrained on Dataset 1 using a binary classification objective (AVP vs. non-AVP) with 15 epochs and a batch size of 64 to learn generalizable sequence representations. Based on this initialization, a transfer learning strategy is adopted, where the pretrained model parameters are transferred to the downstream functional activity prediction task and further fine-tuned using the corresponding functional datasets. This approach enables more precise classification of functional activities. By leveraging transfer learning, the model benefits from knowledge acquired in the pretraining stage, avoiding the need to train from scratch, which typically requires large amounts of data and may lead to unstable optimization. On the one hand, pretraining allows the model to capture fundamental discriminative features between AVPs and non-AVPs. On the other hand, fine-tuning adapts the model to task-specific differences across virus categories, thereby improving its discriminative ability and generalization performance in multi-class settings. In addition, transfer learning accelerates convergence, enhances training efficiency, and mitigates the impact of limited sample sizes.

To address the presence of sequences that do not belong to the predefined virus families or species in the functional activity dataset, we follow the strategy proposed by Cao et al. [15]. Specifically, AVP samples that fall outside the six target virus families are assigned to an additional category labeled as 6 (with labels of 0 to 5 corresponding to the six known families). Similarly, sequences that do not belong to the eight target virus species are assigned a label of 8 (with labels of 0 to 7 representing the eight predefined species). This unknown class setting prevents the model from forcibly assigning non-target sequences to known categories, thereby reducing false positives. Moreover, incorporating such samples into training allows the model to learn subtle distinctions between target classes and general AVP sequences, improving robustness and classification accuracy in open-set scenarios. This strategy better reflects the inherent distribution of biological data and provides a solid foundation for subsequent multi-task prediction.

Our PMAVP model is implemented in Python (v3.6.13), built upon TensorFlow-GPU (v2.6.2), Keras (v2.6.0), and the Transformers library (v4.11.3). All experiments are conducted on a computing platform equipped with an Intel Xeon Silver 4316 CPU (2.30 GHz) and a single NVIDIA GeForce RTX 4090 GPU with 24 GB of memory, running Ubuntu 20.04 LTS. During training, the Adam optimizer is employed for parameter optimization. For rigorous performance evaluation, the dataset is randomly split into training and independent test sets at a ratio of 4:1, and four-fold cross-validation is additionally conducted to assess model robustness.

### 4.9. Evaluation Metrics

To comprehensively and objectively evaluate the performance of the proposed models in AVP identification tasks, six widely used binary classification metrics were employed. Specifically, accuracy (ACC) was used to measure the overall prediction performance across all samples, while sensitivity (Sen) and specificity (Spe) were adopted to assess the model’s ability to identify positive samples and distinguish negative samples, respectively. Precision (Pre) evaluates the proportion of correctly predicted positive samples among all predicted positives, whereas the F1 score provides a balanced assessment by jointly considering precision and sensitivity, making it particularly informative under imbalanced data distributions. Considering the class imbalance present in the datasets used in this study, the Matthews correlation coefficient (MCC) was further introduced as an evaluation metric. The MCC incorporates all four components of the confusion matrix, including true positives, true negatives, false positives, and false negatives, and is therefore regarded as a robust indicator for imbalanced classification problems [33]. In addition, to further assess model performance under different decision thresholds, the area under the receiver operating characteristic curve (AUC) and the area under the precision–recall curve (PRAUC) [34] were also adopted. AUC measures the overall discriminative capability of the model, whereas PRAUC is more suitable for evaluating performance in scenarios with relatively few positive samples. The detailed formulations of these evaluation metrics are given as follows:

(5) $$ACC=\frac{TP+TN}{TP+TN+FP+FN}$$

(6) $$Spe=\frac{TN}{TN+FP}$$

(7) $$Sen=\frac{TP}{TP+FN}$$

(8) $$Precision=\frac{TP}{TP+FP}$$

(9) $$F1\text{-}score=\frac{2Precision\times Recall}{Precision+Recall}$$

(10) $$MCC=\frac{TP\times TN-FP\times FN}{\sqrt{\left(TP+FN)(TP+FP)(TN+FN)(TN+FP\right)}}$$

where TP, TN, FP, and FN denote the numbers of true positives, true negatives, false positives, and false negatives, respectively.

We used four multi-class evaluation metrics—namely, accuracy, macro-precision (MacroP), macro-recall (MacroR), and macro-F1 score (MacroF)—to assess the overall performance and classification robustness of the model for AVP functional activity prediction task. Specifically, accuracy measures the overall classification accuracy across all categories and provides an intuitive summary of model performance. MacroP and MacroR evaluate the precision and recall of each functional category, respectively, thereby enabling effective assessment of whether the model exhibits high false-positive or false-negative rates for specific virus families or species. As a key metric for multi-class classification, MacroF is calculated by averaging the F1 scores across all categories, assigning equal importance to each functional activity class. Consequently, it provides an objective evaluation of the model’s balance under imbalanced class distributions and prevents prediction bias toward high-frequency categories, making it an important indicator of model robustness [35]. The formulations of these metrics are given as follows:

(11) $$Accuracy=\frac{1}{n}\sum_{i=1}^{n}d(f\left(x_{i}\right)=y_{i})$$

(12) $$MacroR=\frac{1}{k}\sum_{i=1}^{k}\frac{TP_{i}}{TP_{i}+{FN}_{i}}$$

(13) $$MacroP=\frac{1}{k}\sum_{i=1}^{k}\frac{TP_{i}}{TP_{i}+{FP}_{i}}$$

(14) $$MacroF=\frac{1}{k}\sum_{i=1}^{k}\frac{2{Precision}_{i}\times {Recall}_{i}}{{Precision}_{i}\times {Recall}_{i}}$$

where n denotes the total number of samples, d represents the indicator function, $f\left(x_{i}\right)$ represents the predicted label generated by the model, and $y_{i}$ represents the true label of sample $x_{i}$. Furthermore, k denotes the total number of classes, and $TP_{i}$, ${FP}_{i}$, and ${FN}_{i}$ represent the numbers of true positives, false positives, and false negatives for class i, respectively.

## Figures and Tables

**Table 1:** Summary of PMAVP model variants.

Model	Architecture
PMAVP-w/oMamba	Removal of the Mamba module
PMAVP-w/oMax	Removal of the global max pooling layer
PMAVP-w/oAvg	Removal of the global average pooling layer
PMAVP-w/oFocal	Replacement of Focal Loss with the standard cross-entropy loss function
PMAVP-w/oPretrain	Removal of the transfer learning strategy for functional activity prediction

**Table 2:** Performance comparison of PMAVP and two existing methods for AVP (a) virus family and (b) virus species prediction using four-fold cross-validation on the dataset.

Method	Accuracy	MacroP	MacroR	MacroF	Mean
(a) Virus family					
PMAVP	0.833 ± 0.006	0.859 ± 0.023	0.785 ± 0.011	0.813 ± 0.017	0.823 ± 0.009
FFMAVP	0.833 ± 0.014	0.832 ± 0.025	0.793 ± 0.015	0.808 ± 0.016	0.817 ± 0.014
AVPIden	0.670 ± 0.025	0.658 ± 0.024	0.726 ± 0.04	0.668 ± 0.029	0.680 ± 0.028
(b) Virus species					
PMAVP	0.823 ± 0.008	0.867 ± 0.019	0.778 ± 0.017	0.808 ± 0.011	0.819 ± 0.008
FFMAVP	0.829 ± 0.015	0.859 ± 0.015	0.786 ± 0.018	0.812 ± 0.016	0.821 ± 0.014
AVPIden	0.749 ± 0.021	0.765 ± 0.014	0.835 ± 0.031	0.773 ± 0.018	0.781 ± 0.019

Note: Performance is shown as mean ± standard deviation.

**Table 3:** Overview of datasets used in this study.

Dataset	Category	Number	Reference
Dataset 1	AVP	2762	[15]
	non-AVP	10,089	
Dataset 2	AVP	600	[16]
	non-AVP	444	
Dataset 3	AVP	600	[16]
	non-AVP	599	

**Table 4:** Optimal hyperparameters of PMAVP.

Hyperparameter	Value
Activation function	ReLU & Softmax
Optimizer	Adam
Learning rate	0.0001
Fully Connected layer	Unit = 1024, 512, 256
Dropout	Dataset 1: γ=1,α=0.75
	Dataset 2: γ=2,α=0.3
	Dataset 3: γ=2,α=0.5

**Table 5:** Optimal hyperparameters of the Mamba module.

Hyperparameter	Value
Activation function	Swish
Depthwise Conv1D	Filter = 2048, kernel size = 4, groups = 2048
Input Projection (linear)	Unit = 4096
State Projection (linear)	Unit = 2048
Output Projection (linear)	Unit = 1024
Dropout	0.1

## Data Availability

The original contributions presented in the study are included in the article. Further inquiries can be directed to the corresponding author.
